# The Prognostic Significance of HALP Index for Colon Cancer Patients in a Hispanic-Based Population

**DOI:** 10.1155/2022/4324635

**Published:** 2022-11-23

**Authors:** German Calderillo Ruiz, Horacio Lopez Basave, Rafael Sebastian Vazquez Renteria, Alison Castillo Morales, Alberto Guijosa, Carolina Castillo Morales, Marytere Herrera, Consuelo Diaz, Ezequiel Vazquez Cortes, Erika Ruiz-Garcia, Wendy R. Munoz Montano

**Affiliations:** ^1^Gastrointestinal Oncology Unit, Instituto Nacional de Cancerologia, Mexico, Mexico; ^2^Universidad Nacional Autonoma de Mexico, Mexico, Mexico; ^3^School of Medicine, Benemerita Universidad Autonoma de Puebla, Puebla, Mexico; ^4^School of Medicine, Universidad Panamericana, Mexico, Mexico

## Abstract

**Background:**

Survival and recurrence rates following locoregional colon cancer surgical resection are highly variable. Currently used tools to assess patient risk are still imperfect. In the present work, we evaluate, for the first time, the prognostic value of the recently developed HALP (hemoglobin, albumin, lymphocyte, and platelet) index in Hispanic colon cancer patients. *Patients and Methods*. We conducted a retrospective cohort study in Mexican patients with a nonmetastatic colon cancer diagnosis who underwent surgical resection. We determined the preoperative HALP score optimal cut-off value by using the X-tile software. We plotted survival curves using the Kaplan–Meier method and performed a multivariate Cox regression analysis to explore the association of preoperative HALP score with two primary endpoints: overall survival (OS) and disease-free survival (DFS).

**Results:**

We included 640 patients (49.8% female). The optimal HALP cut-off value was 15.0. A low HALP index was statistically significantly associated with a higher TNM stage. Low HALP score was statistically significantly associated with shorter median OS in the Kaplan–Meier analysis (73.5 vs. 84.8 months) and in the multivariate Cox regression analysis (HR = 1.942, 95% CI = 1.647–2.875). There was no significant association between the HALP score and DFS.

**Conclusions:**

Our findings show that the HALP index is an independent factor associated with survival in Hispanic patients, despite recurrence. It seems to reflect both the anatomical extent of the disease and traditionally unaccounted nutritional and inflammatory factors that are significant for prognosis.

## 1. Introduction

Colon cancer is a public health concern. At a global level, colorectal carcinoma is currently the third most incidence of cancer and the second most common cause of cancer death. In Mexico, during 2020 alone, this cancer accounted for 14,901 new cases and 7,755 deaths [1].

Surgery is the mainstay of treatment in locoregional colon cancer and continues to be the only curative modality. However, surgery results in a cure only in approximately 50% of cases, as recurrences following surgical resection remain a major problem and are a frequent cause of eventual death [2, 3].

Colon cancer is characterized by being heterogeneous. Whether recurrence occurs or not is possibly dependent on a myriad of factors that result in different individual risks of recurrence [4]. Some high-risk patients benefit from the addition of adjuvant chemotherapy and strict surveillance, while others do not. The latter group of patients could be spared from significant chemotherapy side effects or bothersome procedures, while the former greatly benefit from these interventions, improving survival rates [4, 5]. Therefore, elucidating prognostic factors is an essential task.

The tumor-node-metastasis (TNM) staging system by the American Joint Committee on Cancer (AJCC) is currently the sturdiest and most frequently used tool for assessing patients' prognosis to guide management in colon cancer [6, 7]. However, this tool still has limitations, as individuals within the same stage have highly variable survival rates, and patients' prognoses across different stages sometimes overlap [7, 8].

Cancer progression and metastasis are not solely dependent on tumor characteristics and anatomic extent [9]. It is well established that both systemic inflammation and nutrition play an important role in prognosis [10]. These two factors are linked to many tumor characteristics, including proliferation, invasion, metastasis, and recurrence [11]. Various inflammatory and nutritional markers have been individually associated with survival outcomes in several types of cancer. Moreover, studies have used a combination of these markers to successfully predict prognosis with the use of a single index; examples include the prognostic nutritional index (PNI), the neutrophil-to-lymphocyte ratio (NLR), and the platelet-to-lymphocyte ratio (PLR) [12].

In 2015, Chen et al. developed the novel HALP index, which utilized preoperative hemoglobin, albumin, lymphocyte, and platelet levels to assess gastric cancer patient's prognosis [13]. Shortly after, Jiang et al. studied the prognostic value of this index in colorectal cancer, finding superb results. Their study showed that patients with lower HALP scores had an increased risk of death and cancer-related death, with lower overall survival and cancer-specific survival. All the aforementioned associations were statistically significant and independent of other factors in the multivariate analysis, both in the training and validation sets [14].

Ever since, the HALP index has been validated in several types of cancer, including bladder [15], esophageal [16], lung [11], prostate [9], pancreatic [17], and renal [12]. In these studies, the index has shown to have a clearly superior prognostic value compared to individual markers and other prognostic indexes (PNI, PLR, and NLR) [9, 12, 13, 15–17]. In addition, combining this index with other parameters was reported to be more accurate than TNM staging at predicting patients' prognoses [12, 13].

Given that the HALP index is an easily reproducible tool, which utilizes widely-available routinary lab values, its adoption in clinical practice could significantly benefit developing countries, such as those in Latin America. Unfortunately, so far, this index has not been validated for Hispanic populations, where colon cancer epidemiological and prognostic characteristics are distinct [18]. A formal validation is needed to implement the HALP index in clinical practice.

In the present work, we aim to evaluate the prognostic value of the HALP index for Hispanic colon cancer patients in one of Mexico's largest oncological centers.

## 2. Patients and Methods

### 2.1. Study Design

We conducted a retrospective cohort study in Mexican colon cancer patients without metastatic disease. We explored the association of the HALP index with our two primary endpoints: overall survival (OS) and disease-free survival (DFS). This study was approved by the Institutional Review Board (IRB) at Mexico's National Cancer Institute (No. 2021/143). Written informed consent from patients was waived due to the retrospective nature of this study.

### 2.2. Patients

All patients with a nonmetastatic colon cancer diagnosis who underwent surgical resection at Mexico's National Institute of Cancer, between January 2008 and January 2020, were retrospectively screened for inclusion. We included patients with histologically confirmed stage I–III primary colonic adenocarcinoma who were subjected to radical surgery for the primary tumor. We excluded patients who had been previously treated for cancer, had recurrent or metastatic disease, synchronous malignancies, hereditary colon cancer, underwent other types of surgeries (local excision or palliative surgery), and/or had missing information needed to calculate the HALP index.

### 2.3. Data Collection

Clinical and pathological variables of all included patients were collected from electronic medical records. These included age, gender, body mass index (BMI), date of diagnosis, tumor characteristics (location, differentiation grade, presence of lymphovascular/perineural invasion, and staging characteristics), surgical margin status, and information regarding complications. Also, to calculate the HALP index, preoperative serum albumin, hemoglobin, lymphocyte, and platelet values were collected for all patients. All patients were staged according to the 7th edition AJCC TNM staging system [6]. Information regarding patients' vital status and disease recurrence was collected in order to assess our primary endpoints (OS and DFS). Patients were followed-up regularly (every two weeks while receiving chemotherapy and every two months otherwise). Follow-up care included clinical examinations, laboratory testing, and imaging (TAC, PET-CT), according to international guidelines. Follow-up for each patient began at the date of diagnosis and continued until (1) loss of follow-up, (2) death, or (3) last visit before the cut-off date (January 2020).

### 2.4. Statistical Analysis

HALP index was calculated with the following formula: hemoglobin level (g/L) × albumin level (g/L) × lymphocyte (/L)/platelet count (/L) [13]. The optimal cut-off value for HALP was determined by using the X-tile software (Version 3.6.1, Yale University, USA) [19]. The cut-off with the lowest *p*-value calculated from the chi-squared test for OS was selected and patients were classified as having either low or high HALP. The optimal cut-off value was first determined for the entire cohort, and later it was identified independently within two subgroups (patients who underwent emergency surgery vs. patients who underwent elective surgery), in order to validate our obtained value.

For the analysis, quantitative variables were converted into categories (groups). Age was grouped according to three categories (younger, middle-aged, and senior adults) and BMI was grouped according to nutritional status categories. Frequencies and proportions were reported for all collected variables. Chi-squared test was used to detect associations between the collected variables and the dichotomized HALP index.

The Kaplan–Meier method was used to calculate the median OS and DFS for each variable and plot survival curves. A log-rank test was used to compare survival between groups. Additionally, multivariate Cox regression analysis was performed to identify associations between each variable and survival; hazard ratios (HRs) and the respective 95% confidence intervals (CIs) were calculated. Only statistically significant variables in the univariate analysis were included in the subsequent multivariate analyses. All statistical analyses were performed with SPSS software (SPSS 19.0, IBM, Chicago, IL, USA). A two-tailed *p* value of <0.05 was considered statistically significant.

## 3. Results

### 3.1. Patient Characteristics

893 patient records were screened for inclusion. Ultimately, 640 patients were deemed eligible and included in our final sample.

Among the 640 patients (50.2% males and 49.8% females), the most prevalent age group was between 40 and 70 years of age (middle-aged adults), which represented 66.3% of the sample. Pathological TNM (pTNM) stages II and III represented most of our population (43.1% and 45.2%, respectively). Histologically, tumors were more often moderately (56.6%) and poorly (24.5%) differentiated. As for the surgical procedure, in most cases (85.2%), an adequate lymphadenectomy was achieved (12 or more resected lymph nodes). Nearly all resected primary tumor specimens (97.8%) had microscopically negative surgical margins (R0). Only a few patients had postoperative complications (13.9%). Most surgeries were elective (78.9%). However, 78 patients (12.2%) had colon cancer obstruction, and 57 (8.9%) had perforation, necessitating emergent surgery. All clinicopathological characteristics can be reviewed in Table 1.

### 3.2. Cut-Off Value Determination

**Figure 1:**
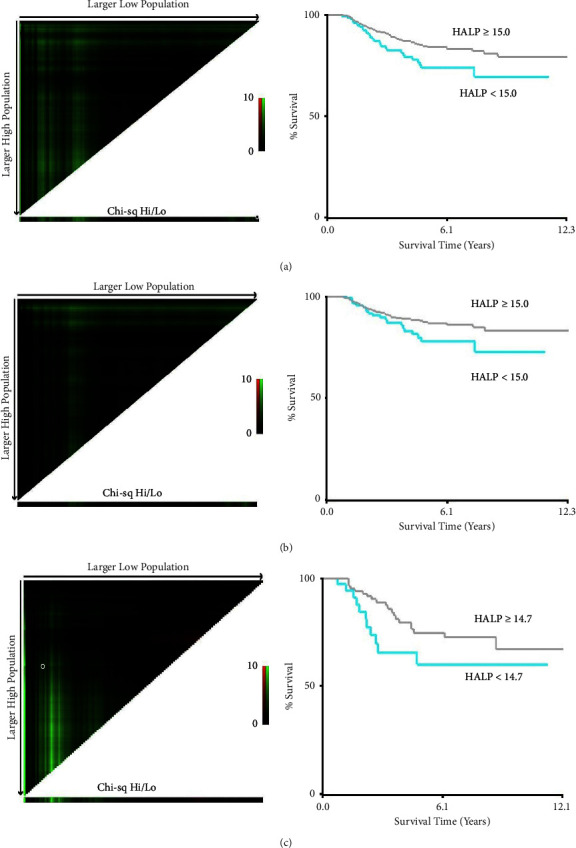
Cut-off values for HALP by X-tile software. (a) The optimal cut-off value for the entire cohort was 15.0. (b) The optimal cut-off value for the subgroup of patients who underwent elective surgery was 15.0. (c) The optimal cut-off value for the subgroup of patients who underwent elective surgery was 14.7.

The optimal cut-off value in the entire cohort for HALP was set at 15.0, and with this dividing point, patients were classified as having either a low or high HALP index. The optimal cut-off points for the two evaluated subgroups, patients who underwent elective surgery vs. those who had emergency surgery, were 15.0 and 14.7, respectively. Given their similarity to the cut-off value obtained for the whole cohort, this value (15.0) was concluded to be a valid cut-off point. X-Tile results and plots are displayed in Figure 1.

### 3.3. HALP Association with Clinicopathological Characteristics

HALP index was statistically significantly associated with pTNM stage, colon cancer location, BMI, tumor differentiation degree, and achievement of adequate lymphadenectomy (12 or more resected lymph nodes).

### 3.4. HALP and Clinicopathological Characteristics Association with Survival

**Figure 2:**
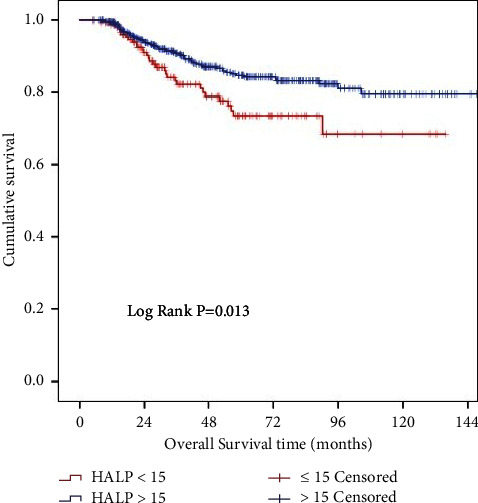
Kaplan–Meier curves for overall survival (OS) according to HALP.

The median follow-up time, OS, and DFS were 46.39, 51.9, and 47.9 months, respectively. In the (univariate) Kaplan–Meier analysis, low HALP score was significantly associated with shorter median OS (73.5 vs. 84.8 months; log-rank test *p*=0.013; Figure 2), as were advanced pTNM stage, poorly differentiated tumors, lymphovascular invasion, perineural invasion, presence of cancer obstruction, *N* positive disease, and positive surgical margins. Multivariate Cox regression analysis established that a low HALP score is an independent factor associated with shorter OS (HR = 1.942, 95% CI = 1.647–2.875; *p*=0.031). All variables' association with OS can be reviewed in Table 2.

**Figure 3:**
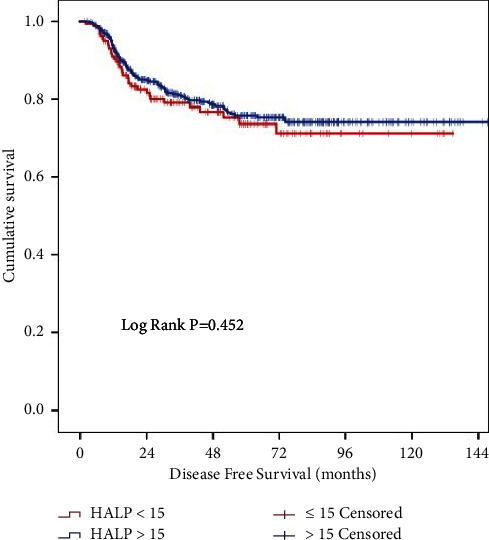
Kaplan–Meier curves for disease-free survival (DFS) according to HALP.

There was no statistically significant association, in the univariate (Figure 3) or multivariate analyses, between HALP score and DFS. The only statistically significant variable associated with DFS in the multivariate analysis was cancer perforation (HR = 1.692, 95% CI = 0.986–2.902; *p*=0.046). All variables' association with DFS can be reviewed in Table 3.

## 4. Discussion

### 4.1. Biological and Clinical Significance of the HALP Index

The HALP index has been found to be a reliable marker for prognosis in cancer patients. It utilizes individually-validated preoperative markers and computes them into a single value, able to identify patients with a higher risk, utilizing the following formula: hemoglobin (g/L) × albumin (g/L) × lymphocytes (/L)/platelets (/L) [13]. Patients with a low HALP score have been shown to have worse outcomes, both in previously published Asian-based studies and in the present, Hispanic-based, study.

Hemoglobin has been widely validated as a prognostic factor for disease progression and survival in a variety of human cancers. Cancer-related anemia (CRA) is present in over 30% of patients at diagnosis. CRA is a consequence of chronic inflammation and poor nutritional status. Both tumor cells and tumor-reacting immune cells release proinflammatory cytokines that directly alter the hematopoietic environment and deteriorate nutritional status, further worsening anemia.

Patients with advanced cancer have a more intense and prolonged inflammatory and nutritiously impoverished state. Consequently, hemoglobin levels serve as an indicator of the degree of disease progression [20]. Moreover, anemia-generated hypoxia in the tumor microenvironment increases the tumor's proliferative and metastatic potential. Anemia has been directly linked to treatment resistance and aggressive disease [21].

Since anemia is an independent risk factor for perioperative morbidity and mortality, hemoglobin levels are a commonly assessed value in surgical patients [22]. In the context of colorectal tumor surgical resection, anemia has been well-validated as a prognostic factor associated with decreased OS and DFS [21].

Serum albumin is one of the most widely used markers to assess nutritional status [23]. Malnutrition in cancer patients derives from cancer's catabolic state and inflammation-induced anorexia. It results in metabolic alterations and muscle wasting, which are directly linked to an increased risk of chemotherapy toxicity, postoperative complications, and death [24]. Concomitantly, albumin is a main negative acute phase reactant. Similarly to hemoglobin, its decay reflects the systemic inflammation produced by cancer [25]. Albumin is also known to stabilize cell growth, promote DNA repair and have antioxidant properties [26]. *In vitro*, albumin has been shown to suppress tumor proliferation [27]. However, this effect may vary *in vivo* depending on the stage the tumor is at, so further studies are needed to elucidate the direct effect of albumin on tumor progression. Clinically, albumin levels are associated with the development of surgical complications and have been well-defined as a long-term survival predictor [26]. Particularly, hypoalbuminemia is associated with wound healing and anastomotic complications [28].

Lymphocytes play an important role in the immune response toward malignant cells. Through both humoral and cellular immune responses, lymphocytes are able to limit tumor growth and metastatic potential. Lymphocyte count and tumor-infiltrating lymphocytes are thought to be indicators of the host's ability to mount an effective immune response to cancer [29]. In colorectal cancer, lymphopenia has been found to be associated with decreased chemotherapy effectiveness and lower overall survival [30, 31].

In contrast with the aforementioned factors, low platelet count has been consistently related to a *better* prognosis. A cohort that included 112,231 cancer patients found that a high platelet count was associated with an increased rate of cancer-specific death [32]. Platelets can release a variety of cytokines, growth factors, and proangiogenic molecules which directly induce tumorigenesis, cancer proliferation, and metastasis. In addition, platelets are able to engulf tumor cells, aiding in cancer immune evasion and further promoting uncontrollable growth and dissemination [33]. Colon cancer patients with elevated platelet counts have shorter OS and DFS [34].

The HALP index efficiently compiles the aforementioned marker values into a joint score, to bolster their independent prognostic effect. This way, a single and practical value is put forth with the aim of reaching the required predictive value for implementation in clinical practice.

### 4.2. Key Results

In our study, the HALP index was found to be independently associated with overall survival. Having a low HALP score nearly doubled the hazard of death (HR = 1.942, 95% CI = 1.647–2.875; *p*=0.031). The HALP index represented the third strongest variable associated with OS, right after the well-defined and widely implemented variables: TNM stage (Stage II vs. Stage III) and surgical margin status (R0 vs. R1/R2).

We did not find a statistically significant association between the HALP index and DFS. This could be attributable to the fact that DFS is largely dependent on other variables that were not included in our analysis, such as receiving adequate adjuvant chemotherapy. It should be noted that a statistically significant association was not found between the TNM stage and DFS either, which gives us further reason to believe that other factors, which were not considered in our analysis, played an important role in determining DFS.

It should be borne in mind that the association identified here between a high HALP score and longer OS suggests that the HALP index is able to predict long-term survival, despite recurrence.

Our sample size was fairly large and the included patients had clinical characteristics similar to those included in previous colon cancer studies undertaken in Mexico and other Latin-American countries [35–37]. Surgical quality indicators, such as successful lymph node resection and microscopically negative surgical margins, were most often achieved. All this supports the generalizability of results to other Hispanic colon cancer populations where quality standards of treatment are met.

The optimal HALP index cut-off point for our population was lower than that reported in previous studies. Using the X-tile software, we determined that 15.0 was the optimal cut-off value, while previous study cut-off points ranged from 22.2 to 56.8 [13, 15]. It should be noted that those studies were undertaken in Asian populations. The difference in our cut-off point may be purely incidental, but it could also stem from biological and clinical differences between Asian and Hispanic populations.

In order to validate our cut-off point of 15.0, we independently calculated the optimal cut-off point in two subgroups (patients who underwent elective surgery vs. those who underwent emergency surgery). In these calculations, we obtained nearly identical cut-off values (15.0 and 14.7, respectively), which confirms the validity of this cut-off point in our overall population. Moreover, even though the nutritional and inflammatory status of patients undergoing elective and emergency surgery may differ significantly, our results support the adoption of the same cut-off point for both populations.

Previous studies have reported female gender [12, 13, 15, 17] and older age [11–13, 15] to be associated with lower HALP scores. Reasons for this association include the fact that female patients tend to have lower hemoglobin levels, while older patients tend to have reduced levels of both hemoglobin and albumin [13, 38, 39]. Nevertheless, this association was not seen in our population. In contrast, left colon cancers in our cohort were associated with lower HALP scores, an association that has not been reported elsewhere. Just as in previous reports, our study found the TNM stage to be associated with the HALP score [12, 13, 15–17]. This is consistent with the belief that computed values for HALP calculation are correlated with the degree of disease progression.

### 4.3. Study Limitations

Our study has some potential limitations. It was a single-center study undertaken in a third-level cancer hospital in Mexico City, which might restrict, to some degree, the generalizability of results to other countries and populations. Additionally, the retrospective nature of this study increases the risk of selection bias. Further assessment of the HALP index with our cut-off point in different Hispanic-based populations should be undertaken.

### 4.4. Future Remarks

Since the HALP index is constructed through modifiable lab values, it is reasonable to believe that preoperative interventions could be undertaken in patients with a low HALP to improve outcomes. Efforts to improve some of the individual HALP-included values have already been undertaken [20, 26]. Knowledge gathered on the prognostic value of the HALP index in our population might indicate that potential preoperative interventions could positively influence long-term survival. It should be noted that implementing these interventions might be more feasible for patients who are undergoing elective surgery, where arranging appropriate preoperative evaluations is possible, in contrast to emergency surgeries. Our future studies will be focused on assessing whether preoperative interventions aimed to improve systemic inflammation, hemoglobin levels, and nutritional status have an impact on patient outcomes.

## 5. Conclusion

Overall, our findings suggest that the HALP index is a viable independent preoperative predictor of survival for Hispanic patients with TNM stages I–III who are undergoing primary tumor surgical resection. These study results give us reason to believe that the HALP index reflects both the anatomical extent of the disease, measured through the TNM staging system, and traditionally unaccounted nutritional and inflammatory factors that play a significant role in prognosis. The wide availability of the routinary lab values needed to compute this score, along with the very practical nature of its implementation, make its adoption in clinical practice feasible. We provide a cut-off value that can be used in similar populations to assess prognosis and guide management.

## Figures and Tables

**Table 1:** General characteristics of patients.

Variables	Total (*n* = 640)	HALP		*p* value
		<15.0	≥15.0	
Age group, *n* (%)				0.974
<40	91 (14.2)	67 (14.0)	24 (14.7)	
40–70	424 (66.3)	317 (66.5)	107 (65.6)	
>70	125 (19.5)	93 (19.5)	32 (19.6)	

Gender, *n* (%)				0.482
Female	319 (49.8)	237 (49.7)	82 (50.3)	
Male	321 (50.2)	240 (50.3)	81 (49.7)	

BMI in kg/m^2^, *n* (%)				**<0.001**
<18.5	24 (3.8)	15 (3.1)	9 (5.5)	
18.5–24.9	315 (49.2)	216 (45.3)	99 (60.7)	
25–29.9	206 (32.2)	163 (34.2)	43 (26.4)	
≥30	95 (14.8)	83 (17.4)	12 (7.4)	

pTNM stage, *n* (%)				**0.02**
I	75 (11.7)	8 (4.9)	67 (14.0)	
II	276 (43.1)	84 (51.5)	192 (40.3)	
III	289 (45.2)	71 (43.6)	218 (45.7)	

Tumor differentiation, *n* (%)				**0.006**
Well	121(18.9)	96 (20.1)	25 (15.3)	
Moderate	362 (56.6)	279 (58.5)	83 (50.9)	
Poor	157 (24.5)	102 (21.4)	55 (33.7)	

Tumor location, *n* (%)				**<0.001**
Right colon	322 (50.3)	205 (43.0)	117 (71.8)	
Left colon	318 (49.7)	272 (57.0)	46 (28.2)	

Lymphovascular invasion, *n* (%)				0.183
No	421 (65.8)	319 (66.9)	102 (62.6)	
Yes	219 (34.2)	158 (33.1)	61 (37.4)	

Perineural invasion, *n* (%)				0.385
No	523 (87.1)	388 (81.3)	135 (82.8)	
Yes	117 (18.3)	89 (18.7)	28 (17.2)	

Number of resected lymph nodes, *n* (%)				**<0.001**
<12	95 (14.8)	85 (17.8)	10 (6.1)	
≥12	545 (85.2)	392 (82.2)	153 (93.9)	

Pathology *T* stage, *n* (%)				**<0.001**
T1	22 (3.4)	19 (4)	3 (1.8)	
T2	69 (10.8)	2 (13.4)	5 (3.1)	
T3	380 (59.4)	290 (60.8)	90 (55.2)	
T4	169 (26.4)	104 (21.8)	65 (39.9)	

Pathology *N* stage, *n* (%)				0.372
N0	357 (55.8)	263 (55.1)	94 (57.7)	
N1	164 (25.6)	119 (24.9)	45 (25.6)	
N2	119 (18.6)	95 (19.9)	24 (14.7)	

Surgical margins/type of resection, *n* (%)				0.589
R0	626 (97.8)	465 (97.5)	161 (98.8)	
R1	13 (2.0)	11 (2.3)	2 (1.2)	
R2	1 (0.2)	1 (1.2)	0	

Cancer obstruction, *n* (%)				0.424
No	562 (87.8)	420 (88.1)	142 (87.1)	
Yes	78 (12.2)	57 (11.9)	21 (12.9)	

Cancer perforation, *n* (%)				0.170
No	583 (91.1)	438 (91.8)	145 (89)	
Yes	57 (8.9)	39 (8.2)	18 (11)	

CCI, *n* (%)				0.377
0	180 (21.8)	126 (26.4)	54 (33.1)	
1	152 (23.8)	118 (24.7)	34 (20.9)	
2	129 (20.2)	96 (20.1)	33 (20.2)	
≥3	179 (28.0)	137 (28.7)	42 (25.8)	

Postoperative complications, *n* (%)				0.227
No	551 (86.1)	414 (86.8)	137 (84)	
Yes	89 (13.9)	63 (13.2)	26 (16)	

Adjuvant chemotherapy, *n* (%)				0.146
No	244 (38.1)	188 (39.4)	56 (34.4)	
Yes	396 (61.9)	289 (60.6)	107 (65.6)	

HALP: hemoglobin (g/L) × albumin (g/L) × lymphocytes (/L)/platelets (/L), BMI: body mass index, pTNM: pathological tumor-node-metastasis stage, CCI: Charlson comorbidity index.

**Table 2:** Univariate and multivariate analyses for factors associated with overall survival.

	Total (events)	Median (95% CI)	*p* value	HR (95% CI)	*p* value
Age (yr)			0.43		
<65	455 (62)	83.6 (79.6–87.5)			
≥65	185 (29)	77.3 (69.2–85.3)			

Sex			0.271		
Female	319 (39)	84.9 (80.1–89.6)			
Male	321 (52)	79.4 (74.1–84.6)			

pTNM stage			**<0.001**		**0.001**
I	75 (0)	No events		10.616 (2.480–45.441)	
II	276 (22)	90.6 (86.4–94.7)			
III	289 (69)	68.5 (61.-75.1)			

Tumor differentiation			**<0.001**		0.086
Well and moderate	483 (58)	85.6 (81.8–89.3)		1.227 (0.972–1550)	
Poor	157 (33)	69.0 (59.2–78.8)			

Lymphovascular invasion			**<0.001**		0.188
No	421 (42)	87.7 (83.9–91.4)		1.381 (0.854–2.235)	
Yes	219 (49)	71.0 (63.5–78.4)			

Perineural invasion			**<0.001**		0.115
No	523 (63)	85.0 (81.2–88.7)		1.489 (0.907–2.443)	
Yes	117 (28)	67.6 (56.8–78.3)			

CCI			0.211		
0	180 (23)	85.8 (80.1–91.7)			
≥I	460 (68)	80.3 (75.7–84.8)			

Cancer obstruction			**0.047**		0.110
No	562 (72)	83.7 (79.9–87.4)		1.532 (0.908–2.583)	
Yes	78 (19)	72.4 (61.4–83.3)			

Cancer perforation			0.123		
No	583 (79)	83.1 (79.3–86.8)			
Yes	57 (12)	74.1 (57.1–86.5)			

Number of resected lymph nodes			0.303		
<12	95 (18)	78.4 (69.1–87.6)			
≥12	545 (73)	82.8 (78.8–86.7)			

Pathology N stage			**<0.001**		0.082
*N* negative	357 (23)	92.4 (89.1–95.7)		0.268 (0.061–1.182)	
*N* positive	283 (68)	69.4 (62.9–75.9)			

Surgical margins/type of resection			0.009		***0.041***
R0	626 (86)	82.9 (79.4–86.4)		2.619 (1.039–6.599)	
R1/R2	14 (5)	45.0 (09.5–80.5)			

HALP score			0.013		**0.031**
<15	162 (31)	73.5 (64.9–82.1)		**1.942 (1.647–2.875)**	
≥15.0	477 (60)	84.8 (80.5–89.1)			

HALP: hemoglobin (g/L) × albumin (g/L) × lymphocytes (/L)/platelets (/L), BMI: body mass index, pTNM: pathological tumor-node-metastasis stage, CCI: Charlson comorbidity index.

**Table 3:** Univariate and multivariate analyses for factors associated with disease-free survival.

	Total (events)	Median (95% CI)	*p* value	HR (95% CI)	*p* value
Age (yr)			0.705		
<65	455 (97)	74.3 (69.5–79.0)			
≥65	185 (34)	78.4 (71.5–85.2)			
Sex			0.904		
Female	319 (66)	74.3 (68.6–79.9)			
Male	321 (65)	76.1 (70.8–81.3)			
pTNM stage			**<0.001**		0.136
I	75 (6)	88.1 (78.8–97.3)		1.963 (0.809–4.762)	
II	276 (34)	86.8 (82.3–91.3)			
III	289 (91)	61.0 (54.3–67.6)			
Tumor differentiation			**0.001**		0.125
Well and moderate	483 (89)	78.4 (73.9–82.5)		1.169 (0.958–1.426)	
Poor	157 (42)	65.7 (56.3–75.1)			
Lymphovascular invasion			**<0.001**		0.436
No	421 (67)	81.2 (76.9–85.5)		1.175 (0.783–1.763)	
Yes	219 (64)	63.7 (56.0–71.3)			
Perineural invasion			**<0.001**		0.056
No	523 (93)	79.0 (74.8–83.1)		1.473 (0.961–2.260)	
Yes	117 (38)	56.3 (44.7–67.8)			
CCI			0.616		
0	180 (37)	76.3 (69.4–83.1)			
≥I	460 (94)	74.7 (69.9–79.4)			
Cancer obstruction			0.186		
No	562 (108)	75.9 (71.8–80.0)			
Yes	78 (23)	70.5 (59.7–81.2)			
Cancer perforation			**0.002**		**0.046**
No	583 (111)	77.1 (73.1–81.0)		1.692 (0.986–2.902)	
Yes	57 (20)	56.7 (41.6–71.8)			
Number of resected lymph nodes			0.156		
<12	95 (26)	69.0 (59.-78.9)			
≥12	545 (105)	76.4 (72.0–80.7)			
Pathology *N* stage			**<0.001**		0.793
*N* negative	357 (42)	86.5 (82.4–90.6)		1.150 (0.405–3.2689)	
*N* positive	283 (89)	61.3 (54.6–67.9)			
Surgical margins/type of resection			0.001		0.138
R0	626 (124)	75.9 (71.6–80.2)		1.926 (0.810–4.575)	
R1/R2	14 (7)	47.6 (20.5–74.6)			
HALP score			0.452		0.536
<15	163 (35)	71.2 (62.0–80.4)		1.134 (0.761–1.690)	
≥15.0	477 (96)	75.8 (71.3–80.3)			

HALP: hemoglobin (g/L) × albumin (g/L) × lymphocytes (/L)/platelets (/L), BMI: body mass index, pTNM: pathological tumor-node-metastasis stage, CCI: Charlson comorbidity index.

## Data Availability

All datasets used for the development of this work are available from the corresponding author upon reasonable request.

## Additional Points

*Previous Abstracts.* An abstract of this work was published online for the 2022 ASCO Annual Meeting [40].
